# Associations Between Antibody Fc-Mediated Effector Functions and Long-Term Sequelae in Ebola Virus Survivors

**DOI:** 10.3389/fimmu.2021.682120

**Published:** 2021-05-20

**Authors:** Dominic Paquin-Proulx, Bronwyn M. Gunn, Aljawharah Alrubayyi, Danielle V. Clark, Matthew Creegan, Dohoon Kim, Hannah Kibuuka, Monica Millard, Salim Wakabi, Leigh Anne Eller, Nelson L. Michael, Randal J. Schoepp, Matthew J. Hepburn, Lisa E. Hensley, Merlin L. Robb, Galit Alter, Michael A. Eller

**Affiliations:** ^1^ U.S. Military HIV Research Program, Walter Reed Army Institute of Research, Silver Spring, MD, United States; ^2^ Henry M. Jackson Foundation for the Advancement of Military Medicine, Inc., Bethesda, MD, United States; ^3^ Ragon Institute of MGH, MIT, and Harvard, Cambridge, MA, United States; ^4^ Austere environments Consortium for Enhanced Sepsis Outcomes (ACESO), Henry M. Jackson Foundation for the Advancement of Military Medicine, Inc., Bethesda, MD, United States; ^5^ Makerere University Walter Reed Project, Kampala, Uganda; ^6^ Center for Infectious Disease Research, Walter Reed Army Institute of Research, Silver Spring, MD, United States; ^7^ Diagnostic Systems Division, US Army Medical Research Institute of Infectious Diseases, Fort Detrick, MD, United States; ^8^ Medical Division, US Army Medical Research Institute of Infectious Diseases, Fort Detrick, MD, United States; ^9^ Integrated Research Facility at Fort Detrick, National Institute of Allergy and Infectious Diseases, National Institutes of Health, Frederick, MD, United States; ^10^ Vaccine Research Program, Division of AIDS, National Institute of Allergy and Infectious Diseases, National Institutes of Health, Bethesda, MD, United States

**Keywords:** antibodies, Ebola, Fc-mediated antibody functions, inflammation, long term sequelae

## Abstract

Antibodies that mediate non-neutralizing functions play an important role in the immune response to Ebola virus (EBOV) and are thought to impact disease outcome. EBOV has also been associated with long term sequelae in survivors, however, the extent to which antibodies that mediate non-neutralizing functions are associated with the development of these sequelae is unknown. Here, the presence of antibodies mediating different effector functions and how they relate to long-term sequelae two years after the 2007 Bundibugyo Ebola virus (BDBV) outbreak was investigated. The majority of survivors demonstrated robust antibody effector functional activity and demonstrated persistent polyfunctional antibody profiles to the EBOV glycoprotein (GP) two years after infection. These functions were strongly associated with the levels of GP-specific IgG1. The odds of developing hearing loss, one of the more common sequelae to BDBV was reduced when antibodies mediating antibody dependent cellular phagocytosis (ADCP), antibody dependent complement deposition (ADCD), or activating NK cells (ADNKA) were observed. In addition, hearing loss was associated with increased levels of several pro-inflammatory cytokines and levels of these pro-inflammatory cytokines were associated with lower ADCP. These results are indicating that a skewed antibody profile and persistent inflammation may contribute to long term outcome in survivors of BDBV infection

## Introduction

Ebola virus (EBOV) outbreaks have occurred with an increased frequency since 1994 (1, 2). Four species of EBOV are known to cause EBOV disease (EVD) in humans: Zaire (ZEBOV), Sudan (SUBOV), Tai Forest, and Bundibugyo (BDBV) with fatality rates ranging from 25% to 90% (3). BDBV is known to have caused only two outbreaks with fatality rates of 32% and 36% (4). The Bundibugyo District of Uganda was the site of the first BDBV outbreak in 2007, which resulted in approximately 116 cases and 39 deaths (5, 6). Many EVD survivors have post-Ebola syndrome and continue to experience a range of symptoms. We have previously reported long-term sequelae, such as retro-orbital pain, blurred vision, hearing loss, and arthralgias in survivors of the 2007 BDBV outbreak in Uganda (7). Similar reports from survivors of other species of EBOV infections show a higher risk of arthralgia, myalgia, fatigue, and vision and hearing loss compared to household contacts (8–10). While the causes of post-Ebola syndrome are not clear, direct tissue damage induced by viral persistence and replication and EBOV-induced auto-immunity are two possibilities. In this regard, ZEBOV survivors with long-term sequelae have higher EBOV-specific CD8+ and CD4+ T cells responses (11), potentially pointing to viral persistence, and ZEBOV survivors have long-lasting immune dysfunction characterized by cellular activation, increased levels of inflammatory markers and intestinal tissue damage compared to controls (12). Given that there are now thousands of EVD survivors, understanding the associations between the EBOV immune response and post-Ebola syndrome may help identify potential treatments.

EBOV-specific humoral immune responses are likely necessary for protection from severe disease and most studies measure antibodies that bind the EBOV glycoprotein (GP) as the primary marker for immunogenicity (13) and as surrogate for vaccine efficacy according to the animal rule (14), a process by which animal study can replace human efficacy trials when they are not feasible or ethical. A follow up study of the 100% efficacious rVSV-EBOV found that the majority of vaccinated subjects maintained EBOV specific antibodies two years after vaccination (15). In natural infection, antibodies, including neutralizing antibodies, persist in survivors 10 to 40 years after infection (16–18), and ZEBOV survivors also develop polyfunctional antibodies capable of mediating Fc-effector functions (19). Combinations of monoclonal antibodies against EBOV have been used with success in post-infection treatment in mouse and non-human primate models (18, 20–23), further supporting a role for antibodies in control of EBOV. Animal model results suggest that neutralization, antibody-dependent cell mediated cytotoxicity (ADCC), complement activation, and phagocytosis could be a mechanism by which antibodies confer protection against lethal infection (18, 24–27). Thus, additional studies are needed to better understand how non-neutralizing mechanisms of EBOV antibodies associate with the development or resolution of disease.

In this study, we assessed the biophysical and functional properties of GP specific antibodies and how they relate to long-term sequelae two years after the 2007 BDBV outbreak from 48 survivors and 121 household contacts. We evaluated antibody-dependent cell mediated phagocytosis (ADCP), antibody-dependent neutrophil mediated phagocytosis (ADNP), antibody-dependent complement deposition (ADCD), and antibody dependent NK cell activation (ADNKA). The vast majority of the survivors had BDBV GP specific antibodies mediating two or more functions. Levels of GP-specific IgG1 were strongly associated with all functions. Surprisingly, polyfunctional antibodies were also observed in some of the household contacts. In the survivors, antibodies mediating ADCP, ADCD, or ADNKA were associated with a reduced risk of developing hearing loss, while antibodies mediating ADNP were associated with increased risk of developing joint pain. Furthermore, EBOV survivors with hearing loss had elevated levels of several pro-inflammatory cytokines compared to those without hearing impairment. No similar pattern was found for the other long-term sequelae. Altogether, our results suggest that higher levels of antibody mediating ADCP, ADCD, and ADNKA and lower inflammation are associated with reduced incidence of hearing loss in EBOV survivors.

## Methods

### Subjects

Plasma samples were collected from individuals with documented clinical history of BDBV infection approximately 24 months following survival of the 2007 Bundibugyo outbreak (n=48), household contacts (n=121), or EBOV GP-seronegative individuals from Uganda (n=8). Participants demographics and long-term sequelae have been previously described (7). All subjects provided written consent. This study was approved by the Institutional Review Board of Walter Reed Army Institute of Research in the United States and the Makerere University School of Public Health, Kampala, Uganda.

### Phagocytosis (ADCP, ADNP)

Phagocytosis assays were performed as previously described (28, 29). Briefly, biotinylated GP was conjugated to yellow-green Neutravidin beads (ThermoFisher, Waltham, MA, USA) and incubated with samples for 2 hours prior to adding fresh white blood cells from a healthy control donor peripheral blood (5 x 10^4^ cells/well) for 1 hour (antibody-dependent phagocytosis by neutrophils [ADNP]) or adding THP-1 cells (MilliporeSigma, Burlington, MA, USA) at 2.5 x 10^4^ cells/well for 18 hours [antibody-dependent cellular phagocytosis by monocytes (ADCP)]. Uptake of antibody-bead complexes by cells was determined by flow cytometry, and a phagocytic score was determined: (% fluorescein isothiocyanate [FITC]+ cells) x (gMFI FITC+)/10 000.

### Antibody-Mediated Complement Deposition (ADCD)

ADCD was measure as previously described (30). Biotinylated GP-coated red Neutravidin beads (ThermoFisher) were incubated with heat-inactivated samples. Guinea pig complement (Cedarlane Labs, Burlington, NC, USA) diluted in veronal buffer containing calcium and magnesium (Boston Bioproducts, Ashland, MA, USA) was incubated with antibody-bead complexes for 20 minutes, and C3 deposition onto beads was detected using an anti–guinea pig C3 antibody (MP Biomedicals, Santa Ana, CA, USA) and measured by flow cytometry.

### Antibody-Dependent NK Cell Activation (ADNKA)

Enzyme-linked immunosorbent assay (ELISA) plates were coated with GP antigen (150 ng/well). Wells were washed, blocked, and incubated with samples for 2 hours prior to adding PBMC from one healthy control donors (5 x 10^5^ cells/well) for 6 hours with brefeldin A and GolgiStop (both from BD Biosciences, San Jose, CA, USA). Intracellular cytokine staining to detect IFNγ, TNFα and MIP1β (BD Biosciences) was performed using Fix/Perm (ThermoFisher), and cells were analyzed by flow cytometry.

### Flow Cytometry

Sample were acquired on a 5 laser, 16-parameter BD LSRII SORP flow cytometer. Data were analyzed with FlowJo v.9.9.6 (BD Biosciences).

### Antigen-Specific Antibody Levels

Recombinant Bundibugyo GP minus the transmembrane region (IBT Bioservices, Rockville, MD, USA) was coupled to MagPlex beads (Luminex, Austin TX, USA) according to previously published protocols (31). Samples were diluted and incubated with antigen-coupled beads for 2 hours. Following bead washing, antibody subclasses (IgG1, IgG2, IgG3, IgG4) and isotypes [immunoglobulin M [(IgM), IgA1, IgA2] were detected using PE-labeled secondary antibodies (0.65 μg/mL; Southern Biotech, Birmingham, AL, USA). The geometric mean fluorescence intensity (gMFI) of 30 beads/region was analyzed on a Flexmap 3D instrument (Luminex). A subclass and antigen-specific cutoff was determined by calculating gMFI and standard deviation of the responses measured in BDBV GP-seronegative individuals. The threshold of positivity was defined as gMFI plus five times the standard deviations of the EBOV GP-seronegative individuals.

### Soluble Markers Mesurements

A total of 53 biomarkers were measured from cryopreserved plasma using commercially available kits. Bio-Plex Pro Human Inflammation Panel 1 Luminex kits (BioRad, Hercules, CA, USA) were used to measure IL-27 (p28), gp130/soluble IL-6Rb, IL-34, IL-22, soluble IL-6Ra, interferon-alpha 2 (IFN-α2), IL-26, MMP-2, IL-12 (p40), IL-19, IL-20, IL-29/interferon-lambda 1 (IFN-Λ1), IL-35, IL-32, BAFF/tumor necrosis factor super family (TNFSF) 13B, IL-11, APRIL/TNFSF13, MMP-1, interferon beta (IFNβ), MMP-3, soluble CD163 (sCD163), Pentraxin-3, LIGHT/TNFSF14, TSLP, soluble CD30/tumor necrosis factor receptor super family 8 (TNFRSF8), TWEAK/TNFSF12, Osteocalcin, IL-28a/interferon-lambda 2 (IFN-Λ2), soluble tumor necrosis factor receptor 2 (sTNF-R2), Chitinase3-like1, soluble tumor necrosis factor receptor 1 (sTNF-R1), and Osteopontin. High Sensitivity T-Cell Luminex assay kits (MilliporeSigma) were used to measure ITAC, GM-CSF, Fractalkine, interferon gamma (IFNγ), IL-10, MIP-3a, IL-12 (p70), IL-13, IL-17a, IL-1β, IL-2, IL-21, IL-4, IL-23, IL-5, IL-6, IL-7, IL-8, MIP-1α, MIP-1β, and tumor necrosis factor alpha (TNFα), as previously described (32).

### Statistical Analysis

All statistical analysis was performed using Graph Pad Prism version 8.2.0 for Mac OS (GraphPad Software, La Jolla, CA, USA). Antibody effector function comparisons between BDBV survivors and household contacts were performed using the Mann-Whitney test. Comparisons of the distribution of the number of antibody effector functions between BDBV survivors and household contacts were performed using the Chi-square test. Comparisons of the positivity for a given antibody effector function between BDBV survivors with and without a given sequalae were performed using Fisher’s exact test. Associations were evaluated using Spearman’s rank correlation. Statistically significant associations (p < 0.05) were used to generate networks in Cytoscape (version 3.7.2). Principal component analysis (PCA) was performed with the R basic function, prcomp method, and the results were visualized using R plotly bioconductor package. P-values < 0.05 were considered statistically significant.

## Results

**Figure 1:**
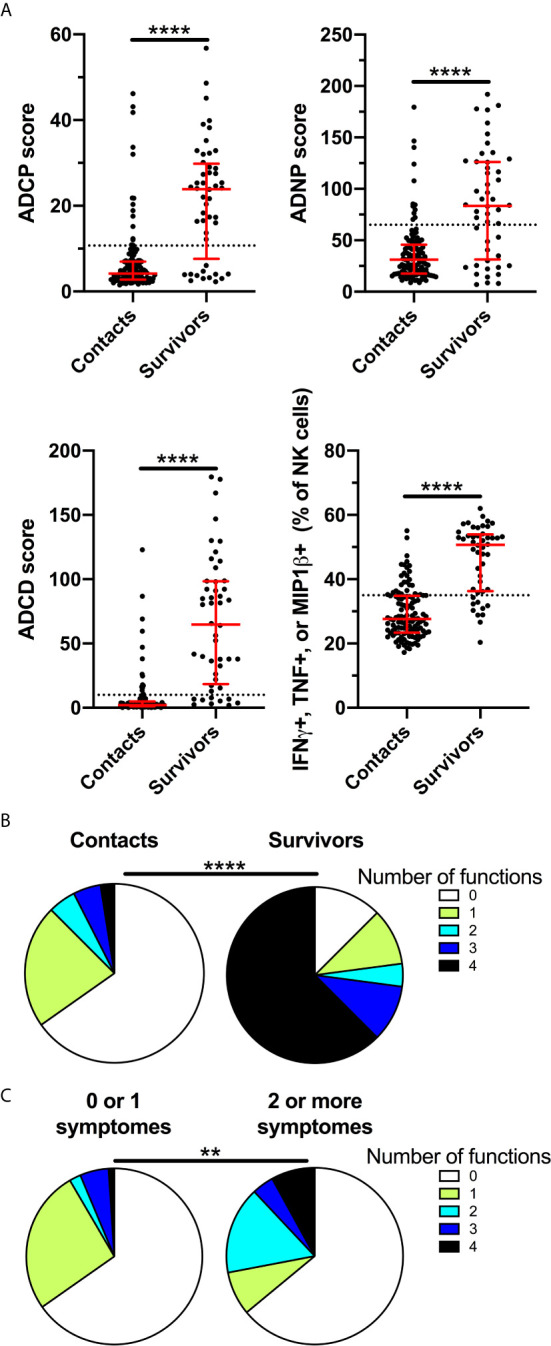
BDBV survivors maintain polyfunctional antibody effector functions for two years. ADCP, ADNP, ADCD, and ADNKA were measured in plasma of BDBV survivors (n=48) and household contacts (n=121) **(A)**. The line and whiskers represent the median and interquartile range respectively. The dotted line represents the cutoff for positivity. Pie chart indicating the number of positive functions for BDBV survivors and household contacts **(B)**. Pie chart indicating the number of positive functions for household contacts with one or less (n=95) and two or more symptoms (n=25) at the time of the outbreak **(C)**. **** represents p values < 0.0001 and ** represents p values < 0.01.

We evaluated the ability of BDBV GP-specific antibodies present in 48 survivors of the 2007 Bundibugyo outbreak and 121 household contacts approximately 24 months after survival to mediate ADCP, ADNP, ADCD, and ADNKA against BDBV GP-coated targets (**Supplementary Figure 1**). Consistent with prior reports regarding the longevity of humoral immunity against Ebola, a majority of survivors had antibodies capable of inducing all functions evaluated (75% ADCP, 64.6% ADNP, 81.3% ADCD, and 79.2% ADNKA) and the magnitude of the response in survivors was significantly higher than that of the household contacts (**Figure 1A**). Of note, positive responses were also observed for a subset of household contacts. Polyfunctionality of anti-Ebola antibodies has been associated with mAb-mediated protection (25), and we found that the majority of survivors (77.1%) had polyfunctional antibodies that could induce 2 or more functions while only a minority of the contacts were (12.5%, **Figure 1B**). A minority (12.5%) of survivors were negative for all functions tested, suggesting that humoral immunity in these survivors was not durable. At the time of the BDBV epidemic, a subset of household contacts displayed some disease symptoms, although these individuals were not confirmed as having EVD (7). Within those contacts that presented with two or more symptoms, polyfunctionality was significantly higher compared to those with fewer symptoms or no symptoms (**Figure 1C**), potentially suggesting that these individuals may represent mild or undiagnosed EVD. Together, these results show that a majority of survivors of BDBV-induced EVD maintain durable polyfunctional antibody effector responses for at least two years after infection.

**Figure 2:**
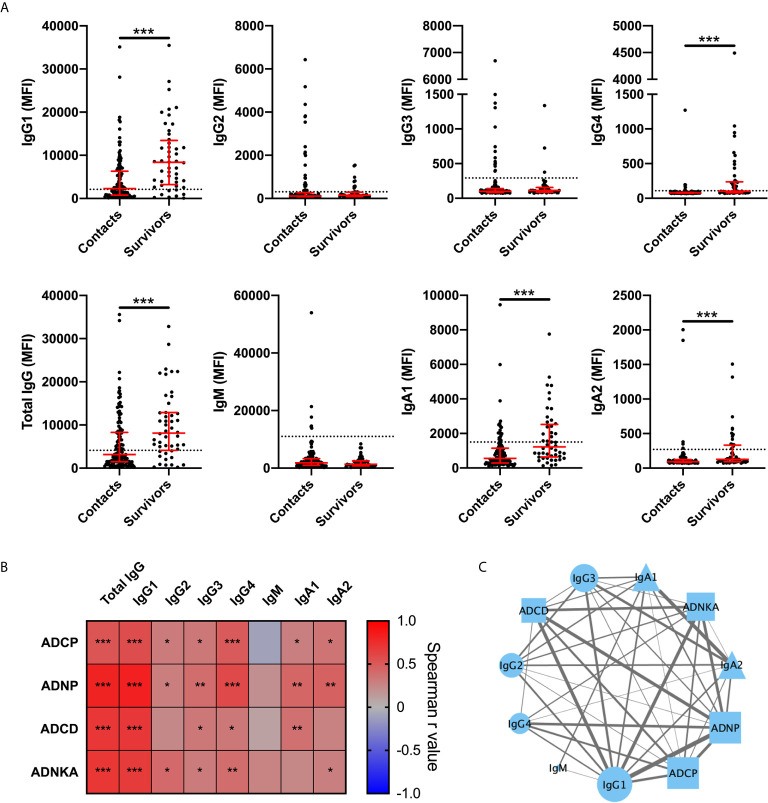
GP specific IgG1 levels are strongly associated with antibody effector functions in BDBV survivors. Levels of BDBV GP specific IgG1, IgG2, IgG3, IgG4, total IgG, IgM, IgA1, and IgA2 in plasma of BDBV survivors (n=48) and household contacts (n=121) **(A)**. The line and whiskers represent the median and interquartile range respectively. The dotted line represents the cutoff for positivity. Heatmap showing the associations between antibody subclasses or isotypes and antibody effector functions **(B)**. Correlation network analysis of statistically significant associations (p < 0.05) between GP-specific functional activity and levels of GP-specific antibody levels **(C)**. The strength of correlation is indicated by the weight of the connecting line and the number of significant associations for each feature is represented by the size of the icon. IgG and IgA subclasses are represented by circles and triangles respectively. IgM is represented by a diamond and Fc-mediated functions are represented by squares. *** represents p values < 0.001, ** represents p values < 0.01, and * represents p values < 0.05.

Next, we determined the relative antibody levels of IgG and IgA subclasses as well as IgM specific for the Bundibugyo GP for BDBV survivors and household contacts. Survivors had significantly higher magnitude of GP-specific IgG1 and IgG4 compared to household contacts (**Figure 2A**). Magnitude of GP-specific IgA1 and IgA2 were also elevated in survivors while levels of IgM, IgG2, and IgG3 were not. All survivors were negative for IgM, consistent with resolution of an infection that occurred two years ago. A subset of the household contacts had levels of GP-specific antibodies (mostly, but not exclusively, IgG1) above the threshold for positivity. Amongst BDBV survivors, IgG1 levels were strongly associated with Fc-mediated effector functions (**Figures 2B, C**). IgG4, and to lower extent IgG2, IgG3, IgA1, and IgA2, were also associated with Fc-mediated effector functions (**Figure 2B**). A network analysis of antibody features demonstrated significant correlations between the levels of GP-specific antibody isotypes and antibody effector functions, revealing a coordinated humoral responses in EVD survivors (**Figure 2C**).

Given the potential protective and pathologic roles of innate immune cells in disease manifestations, we then asked if antibody effector functions were associated with the long-term sequelae that were previously reported in this cohort of BDBV survivors (7). There was a trend for increased incidence of joint pain in the individuals positive for ADNP (odds 3.84; p=0.068, **Table 1**), potentially pointing to an immunopathologic role for neutrophils in sequelae. In contrast, the incidence of hearing loss was reduced in survivors that showed positive responses for ADCP (trending, odds 0.24; p = 0.061), ADNKA (odds 0.16; p = 0.016), or ADCD (odds 0.21; p = 0.048). There was no significant change to the incidence of retro orbital pain, fatigue, or blurred vision based on the Fc-mediated antibody effector functions. Importantly, the total levels of BDBV-specific IgG were not different between survivors with or without long-term sequalae (data not shown), suggesting that a qualitative rather than quantitative features of antibodies tracks with protection from certain EVD-associated sequelae.

**Table 1:** Odds ratio of BDBV survivors to suffer from long term sequelae based on positivity for antibody effector functions.

ADNP		Odds	95% CI	P value
	Joint pain	3.84	1.01-14.2	0.068
	Hearing loss	0.40	0.12-1.41	0.29
	Retro orbital pain	1.20	0.31-4.06	0.99
	Fatigue	1.41	0.44-4.36	0.76
	Blurred vision	3.76	0.98-13.96	0.11
**ADCP**				
	Joint pain	3.57	0.71-17.77	0.17
	Hearing loss	0.24	0.06-0.88	0.061
	Retro orbital pain	0.54	0.14-2.0	0.45
	Fatigue	1.57	0.46-5.22	0.52
	Blurred vision	2.40	0.57-9.16	0.32
**ADNKA**				
	Joint pain	0.27	0.077-1.03	0.13
	**Hearing loss**	**0.016**	**0.041-0.63**	**0.016**
	Retro orbital pain	0.31	0.071-1.34	0.13
	Fatigue	1.53	0.39-6.07	0.72
	Blurred vision	1.70	0.40-6.70	0.72
**ADCD**				
	Joint pain	1.12	0.27-4.58	0.99
	**Hearing loss**	**0.21**	**0.055-0.99**	**0.048**
	Retro orbital pain	0.79	0.18-3.28	0.99
	Fatigue	0.65	0.16-2.61	0.72
	Blurred vision	0.78	0.18-2.89	0.99

P values < 0.05 are indicated bold.

**Figure 3:**
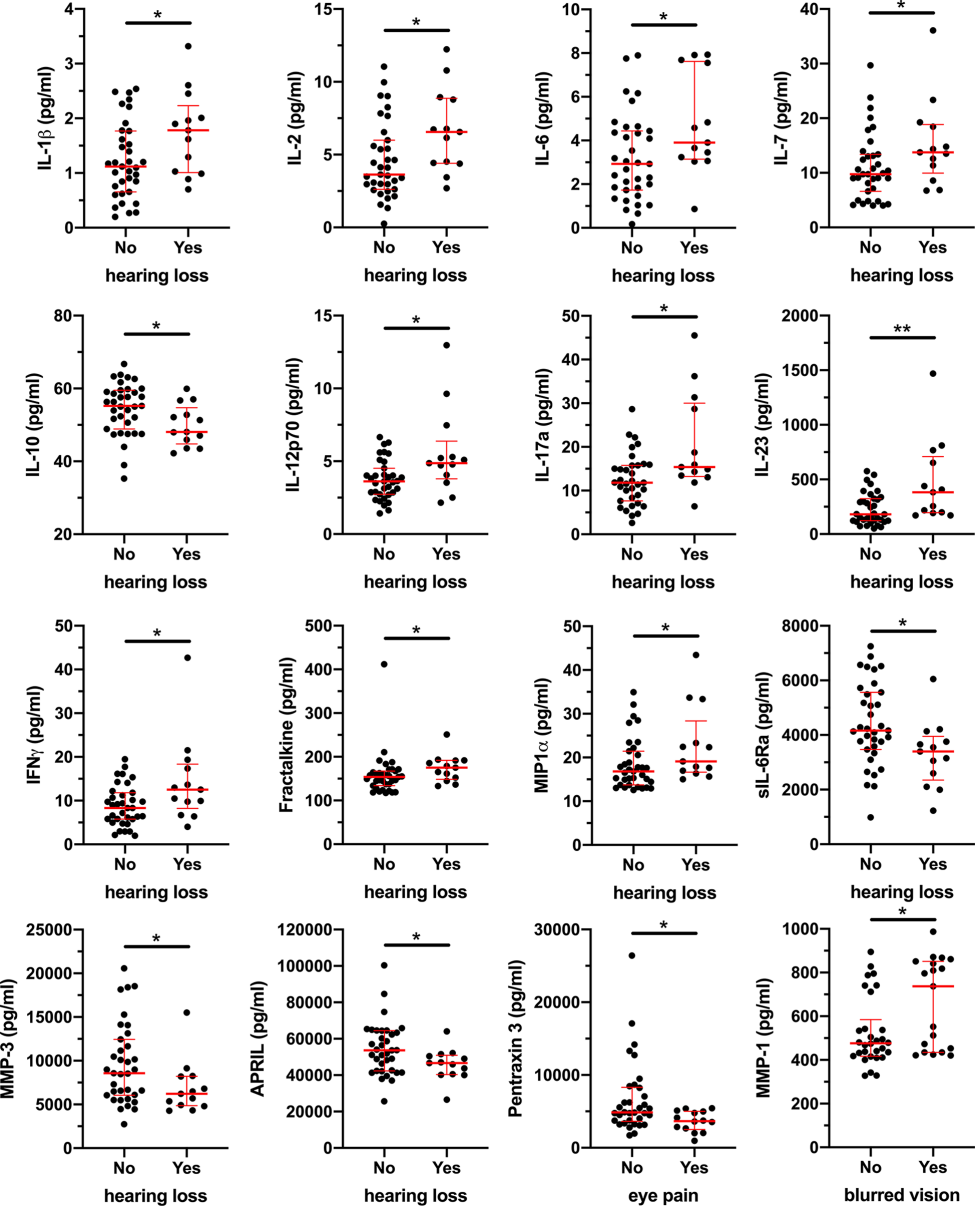
Elevated plasma levels of pro-inflammatory soluble factors in BDBV survivors with hearing loss. Plasma levels of 53 soluble factors were measure by Luminex and results were compared between BDBV survivors with or without long term sequalae. Significant differences are shown for IL-1β, IL-2, IL-6, IL-7, IL-10, IL-12p70, IL-17a, IL-23, IFNγ, Fractalkine, MIP1α, sIL-6Ra, MMP-3, and APRIL for hearing loss as well as Pentraxin 3 and MMP-1 for eye pain and blurred vision respectively. The line and whiskers represent the median and interquartile range respectively. ** represents p values < 0.01 and * represents p values < 0.05.

While the mechanisms underlying development of sequelae is not known, previous studies reported elevated levels of IL-8, TNF, and sCD163 in ZEBOV survivors compared to uninfected subjects (12). Therefore, to evaluate if BDBV survivors are characterized by a pro-inflammatory state, we measured 53 soluble markers associated with inflammation and immune activation in plasma. While we did not observe differences in plasma levels of IL-8, TNF, and sCD163 between BDBV survivors and household contacts, BDBV survivors had significantly lower levels of GM-CSF and sCD30/TNFRSF8 as well as higher levels of APRIL compared to household contacts (**Supplementary Figure 2**). Within the BDBV survivors, hearing loss was marked by increased levels of pro-inflammatory cytokines (IL-1β, IL-2, IL-6, IL-7, IL-12p70, IL-17a, IL-23, and IFNγ) and decreased levels of the anti-inflammatory cytokine IL-10 (**Figure 3**). Moreover, the levels of chemokines Fractalkine and MIP1α were similarly elevated in BDBV survivors with hearing loss while levels of sIL6-Ra, MMP-3, and APRIL were reduced. PCA analysis of the soluble markers indicates that BDBV survivors with hearing loss tend to cluster together but still overlapped with some survivors without hearing loss (**Supplementary Figure 3**). With regard to the other sequelae, Pentraxin 3 levels were lower in BDBV survivors with eye pain and levels of MMP-1 were higher in BDBV survivors with blurred vision (**Figure 3**). Together, these data indicate that BDBV survivors with a subset of sequelae may be chronically inflamed.

**Figure 4:**
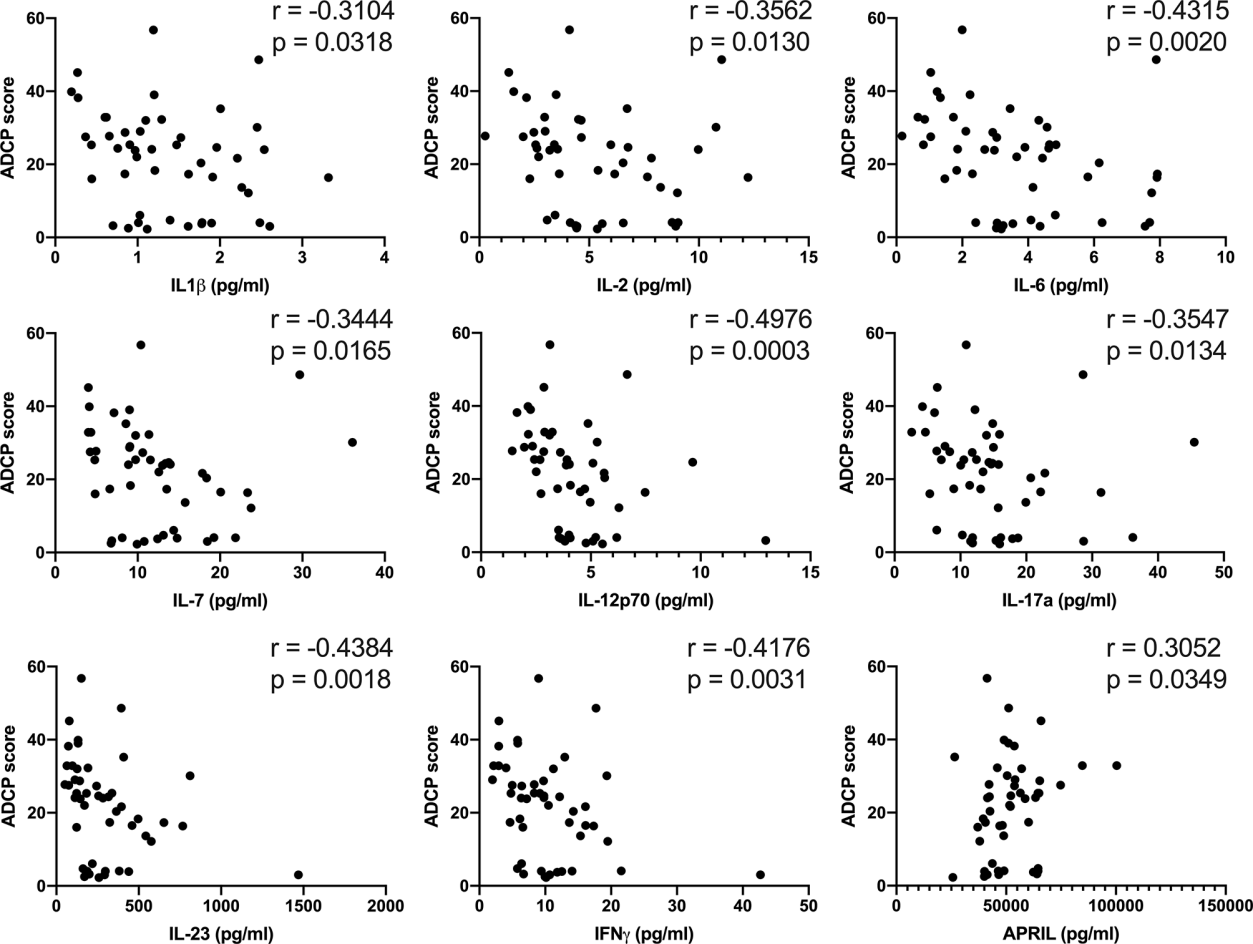
Plasma levels of pro-inflammatory soluble factors in BDBV survivors are inversely associated with ADCP. Associations between the plasma levels of IL-1β, IL-2, IL-6, IL-7, IL-12p70, IL-17a, IL-23, IFNγ, as well as APRIL and ADCP in BDBV survivors.

Persistent inflammation can impact antibody effector function, and thus we performed association analyses to determine if plasma levels of cytokines tracked with antibody effector functions. IL-1β, IL-2, IL-6, IL-7, IL12p70, IL-17a, IL-23, and IFNγ were all inversely associated with ADCP (**Figure 4**), while ADCP was positively associated with plasma levels of APRIL (**Figure 4**). No significant associations were found with ADCD, ADNP, and ADNKA. These results suggest possibility that inflammation in BDBV survivors is associated with lower antibody-phagocytic activity.

## Discussion

In this study, we have demonstrated that BDBV survivors maintain polyfunctional antibody effector activity for two years after infection. This extends findings from a previous report of a cohort from Sierra Leone that showed polyfunctional antibodies six months after infection with ZEBOV (19). In this cohort, both IgG1 and IgA were associated with levels of functional antibodies. Similarly, we found that IgG1, IgA1, and IgA2 levels were increased in this cohort of BDBV survivors compared to household contacts and that Fc-mediated antibody effector functions were strongly associated with GP-specific IgG1 levels. Together, these studies indicate that the development of durable functional antibodies may be a general feature of humoral immunity in EVD caused by different ebola viruses.

A surprising finding of this study is that a minority of household contacts also had polyfunctional antibody effector functions. Polyfunctional responses were more frequent in the contacts that presented two or more symptoms at the time of the outbreak, indicating that some of them might have a milder or subclinical form of the disease, as suggested by other studies (33–35). Alternatively, it has also been documented that EBOV seropositivity in parts of Africa, including Uganda, is associated with wild animals contacts (35–37), and thus this baseline seropositivity may also contribute to the Fc-mediated antibody effector response that we observed in some household contacts.

Our results demonstrate that Fc-mediated effector functions could have a protective role against hearing loss, one of the long-term sequelae previously reported to be associated with BDBV (7). While the mechanisms underlying the development of sequelae in EVD survivors is not known, one possibility is that these functions could have contributed to faster viral clearance during initial infection, and thus limit EBOV-mediated damage. We do not know how early following infection that these antibody functions may have appeared, however longitudinal analysis of EVD survivors from Sierra Leone suggest that antibody functional profiles are consistent for at least 3 years (B. Gunn and G. Alter, unpublished data). Interestingly, the total levels of IgG were not different between the survivors with or without sequelae, consistent with a previous study that also found that binding and neutralizing titers were not associated with long-term sequalae in EBOV survivors (11), together suggesting that antibody effector functionality may be a more important contributor to the development of or protection from sequelae.

In addition to lower levels of antibody-mediated effector functions, hearing loss was characterized by elevated plasma levels of several pro-inflammatory cytokines and lower levels of IL-10. This increase inflammatory state could be caused by viral persistence, increased susceptibility to other infections, or microbial translocation associated with increased damage to the gut barrier integrity, as observed in chronic HIV patients (38). Continued antigen stimulation after resolution of EBOV disease has been suggested to contribute to sustained CD8 T cell activation (39) and to the periodical waxing and waning of antibody levels (40). Furthermore, high levels of pro-inflammatory cytokines were associated with lower ADCP, suggesting that inflammation may impair the development or durability of some humoral responses. Alternatively, high levels of Fc-mediated functions may help reduce EBOV-associated inflammation. While previous work reported that several pro-inflammatory markers were elevated in the plasma of ZEBOV survivors compared to controls, we did not observe differences in our cohort of BDBV survivors and their household contacts two years following the outbreak. While differences in sampling time may underlie these observation, differences in mortality between BDBV and ZEBOV may also explain this discrepancy (4).

No clinically relevant differences in results from the hematology or clinical chemistry laboratories were initially reported between BDBV survivor and their contacts (7). Similarly, no differences were found between the survivors with or without hearing loss (data not shown). One limitation of this study is small number of BDBV survivor that developed each long-term sequelae.

Other acute viral infections, such as SARS-CoV-2, MERS, and Chikungunya, have been associated with long term symptoms even after viral clearance (41–44). Post-acute viral infection syndrome may thus be more common than originally thought and this work may be informative for such other conditions.

In conclusion, our results suggest that antibodies mediating ADCP, ADCD, and ADNKA are associated with lower incidence of hearing loss in BDBV survivors and that hearing loss is characterized by high levels of several pro-inflammatory cytokines, warranting further investigation into the role of antibodies in post-Ebola syndrome.

## Data Availability Statement

The original contributions presented in the study are included in the article/**Supplementary Material**. Further inquiries can be directed to the corresponding author.

## Ethics Statement

The studies involving human participants were reviewed and approved by Institutional Review Board of Walter Reed Army Institute of Research in the United States and the Makerere University School of Public Health, Kampala, Uganda. The patients/participants provided their written informed consent to participate in this study.

## Author Contributions

Performed experiments: DP-P, BG, AA, and MC. Analyzed data: DP-P, BG, AA, MC, and DK. Study Design: GA and ME. Oversaw RV281 cohort: DC, HK, MM, SW, LE, NM, RS, MH, LH, and MR. Wrote the manuscript: DP-P and ME. All authors contributed to the article and approved the submitted version.

## Funding

This work was funded by cooperative agreement (W81XWH-18-2-0040) between the Henry M. Jackson Foundation for the Advancement of Military Medicine, Inc., and the U.S. Department of Defense (DOD). The funding source had no role in the study design; collection, analysis, and interpretation of data; writing of the manuscript; or in the decision to publish.

## Disclaimer

The content of this manuscript is solely the responsibility of the authors and does not necessarily represent the official views of any of the institutions mentioned above, the U.S. Department of the Army or the U.S. Department of Defense, the Henry M. Jackson Foundation for the Advancement of Military Medicine, the National Institutes of Health, the Department of Health and Human Services, or the United States government. The investigators have adhered to the policies for protection of human participants as prescribed in AR-70-25. This article was prepared while Michael A. Eller was employed at Henry M. Jackson Foundation for the Advancement of Military Medicine for the U.S. Military HIV Research Program.

## Conflict of Interest

GA is a founder of SeroMyx Systems Inc.

The remaining authors declare that the research was conducted in the absence of any commercial or financial relationships that could be construed as a potential conflict of interest.

## Abbreviations

EBOV, Ebola virus; ZEBOV, Zaire Ebola virus (ZEBOV); SUBOV, Sudan Ebola virus; BDBV, Bundibugyo Ebola virus; GP, glycoprotein; ADCC, antibody-dependent cell mediated cytotoxicity; ADCP, antibody-dependent cell mediated phagocytosis; ADNP, antibody-dependent neutrophil mediated phagocytosis; ADCD, antibody-dependent complement deposition; ADNKA, antibody dependent NK cell activation; sCD163, soluble CD163.

## References

[1] Roddy P. A Call to Action to Enhance Filovirus Disease Outbreak Preparedness and Response. Viruses (2014) 6(10):3699–718. 10.3390/v6103699 PMC4213557 25271875

[2] Polonsky JA Wamala JF de Clerck H Van Herp M Sprecher A Porten K. Emerging Filoviral Disease in Uganda: Proposed Explanations and Research Directions. Am J Trop Med Hyg (2014) 90(5):790–3. 10.4269/ajtmh.13-0374 PMC4015565 24515940

[3] Feldmann H Geisbert TW. Ebola Haemorrhagic Fever. Lancet (2011) 377(9768):849–62. 10.1016/S0140-6736(10)60667-8 PMC3406178 21084112

[4] Rojas M Monsalve DM Pacheco Y Acosta-Ampudia Y Ramirez-Santana C Ansari AA. Ebola Virus Disease: An Emerging and Re-Emerging Viral Threat. J Autoimmun (2020) 106:102375. 10.1016/j.jaut.2019.102375 31806422

[5] Wamala JF Lukwago L Malimbo M Nguku P Yoti Z Musenero M. Ebola Hemorrhagic Fever Associated With Novel Virus Strain, Uganda, 2007-2008. Emerg Infect Dis (2010) 16(7):1087–92. 10.3201/eid1607.091525 PMC3321896 20587179

[6] Towner JS Sealy TK Khristova ML Albarino CG Conlan S Reeder SA. Newly Discovered Ebola Virus Associated With Hemorrhagic Fever Outbreak in Uganda. PloS Pathog (2008) 4(11):e1000212. 10.1371/journal.ppat.1000212 19023410 PMC2581435

[7] Clark DV Kibuuka H Millard M Wakabi S Lukwago L Taylor A. Long-Term Sequelae After Ebola Virus Disease in Bundibugyo, Uganda: A Retrospective Cohort Study. Lancet Infect Dis (2015) 15(8):905–12. 10.1016/S1473-3099(15)70152-0 25910637

[8] Bwaka MA Bonnet MJ Calain P Colebunders R De Roo A Guimard Y. Ebola Hemorrhagic Fever in Kikwit, Democratic Republic of the Congo: Clinical Observations in 103 Patients. J Infect Dis (1999) 179 Suppl 1:S1–7. 10.1086/514308 9988155

[9] Rowe AK Bertolli J Khan AS Mukunu R Muyembe-Tamfum JJ Bressler D. Clinical, Virologic, and Immunologic Follow-Up of Convalescent Ebola Hemorrhagic Fever Patients and Their Household Contacts, Kikwit, Democratic Republic of the Congo. Commission de Lutte contre les Epidemies a Kikwit. J Infect Dis (1999) 179 Suppl 1:S28–35. 10.1086/514318 9988162

[10] Tozay S Fischer WA Wohl DA Kilpatrick K Zou F Reeves E. Long-Term Complications of Ebola Virus Disease: Prevalence and Predictors of Major Symptoms and the Role of Inflammation. Clin Infect Dis (2020) 71(7):1749–55. 10.1093/cid/ciz1062 PMC7755089 31693114

[11] LaVergne SM Sakabe S Kanneh L Momoh M Al-Hassan F Yilah M. Ebola-Specific CD8+ and CD4+ T-Cell Responses in Sierra Leonean Ebola Virus Survivors With or Without Post-Ebola Sequelae. J Infect Dis (2020) 222(9):1488–97. 10.1093/infdis/jiaa268 PMC7529037 32436943

[12] Wiedemann A Foucat E Hocini H Lefebvre C Hejblum BP Durand M. Long-Lasting Severe Immune Dysfunction in Ebola Virus Disease Survivors. Nat Commun (2020) 11(1):3730. 10.1038/s41467-020-17489-7 32709840 PMC7381622

[13] Gross L Lhomme E Pasin C Richert L Thiebaut R. Ebola Vaccine Development: Systematic Review of Pre-Clinical and Clinical Studies, and Meta-Analysis of Determinants of Antibody Response Variability After Vaccination. Int J Infect Dis (2018) 74:83–96. 10.1016/j.ijid.2018.06.022 29981944

[14] Sullivan NJ Martin JE Graham BS Nabel GJ. Correlates of Protective Immunity for Ebola Vaccines: Implications for Regulatory Approval by the Animal Rule. Nat Rev Microbiol (2009) 7(5):393–400. 10.1038/nrmicro2129 19369954 PMC7097244

[15] Huttner A Agnandji ST Combescure C Fernandes JF Bache EB Kabwende L. Determinants of Antibody Persistence Across Doses and Continents After Single-Dose rVSV-ZEBOV Vaccination for Ebola Virus Disease: An Observational Cohort Study. Lancet Infect Dis (2018) 18(7):738–48. 10.1016/S1473-3099(18)30165-8 PMC6408946 29627147

[16] Rimoin AW Lu K Bramble MS Steffen I Doshi RH Hoff NA. Ebola Virus Neutralizing Antibodies Detectable in Survivors of Theyambuku, Zaire Outbreak 40 Years After Infection. J Infect Dis (2018) 217(2):223–31. 10.1093/infdis/jix584 PMC5853670 29253164

[17] Natesan M Jensen SM Keasey SL Kamata T Kuehne AI Stonier SW. Human Survivors of Disease Outbreaks Caused by Ebola or Marburg Virus Exhibit Cross-Reactive and Long-Lived Antibody Responses. Clin Vaccine Immunol (2016) 23(8):717–24. 10.1128/CVI.00107-16 PMC4979172 27335383

[18] Corti D Misasi J Mulangu S Stanley DA Kanekiyo M Wollen S. Protective Monotherapy Against Lethal Ebola Virus Infection by a Potently Neutralizing Antibody. Science (2016) 351(6279):1339–42. 10.1126/science.aad5224 26917593

[19] Gunn BM Roy V Karim MM Hartnett JN Suscovich TJ Goba A. Survivors of Ebola Virus Disease Develop Polyfunctional Antibody Responses. J Infect Dis (2020) 221(1):156–61. 10.1093/infdis/jiz364 PMC7184900 31301137

[20] Pascal KE Dudgeon D Trefry JC Anantpadma M Sakurai Y Murin CD. Development of Clinical-Stage Human Monoclonal Antibodies That Treat Advanced Ebola Virus Disease in non-Human Primates. J Infect Dis (2018) 218(5):S612–26. 10.1093/infdis/jiy285 PMC6249601 29860496

[21] Froude JW Herbert AS Pelat T Miethe S Zak SE Brannan JM. Post-Exposure Protection in Mice Against Sudan Virus by a Two Antibody Cocktail. Viruses (2018) 10(6):1–12. 10.3390/v10060286 PMC6024315 29861435

[22] Bornholdt ZA Turner HL Murin CD Li W Sok D Souders CA. Isolation of Potent Neutralizing Antibodies From a Survivor of the 2014 Ebola Virus Outbreak. Science (2016) 351(6277):1078–83. 10.1126/science.aad5788 PMC4900763 26912366

[23] Gilchuk P Mire CE Geisbert JB Agans KN Deer DJ Cross RW. Efficacy of Human Monoclonal Antibody Monotherapy Against Bundibugyo Virus Infection in Nonhuman Primates. J Infect Dis (2018) 218(suppl_5):S565–73. 10.1093/infdis/jiy295 PMC6249568 29982718

[24] Liu Q Fan C Li Q Zhou S Huang W Wang L. Antibody-Dependent-Cellular-Cytotoxicity-Inducing Antibodies Significantly Affect the Post-Exposure Treatment of Ebola Virus Infection. Sci Rep (2017) 7:45552. 10.1038/srep45552 28358050 PMC5372081

[25] Gunn BM Yu WH Karim MM Brannan JM Herbert AS Wec AZ. A Role for Fc Function in Therapeutic Monoclonal Antibody-Mediated Protection Against Ebola Virus. Cell Host Microbe (2018) 24(2):221–33.e5. 10.1016/j.chom.2018.07.009 30092199 PMC6298217

[26] Saphire EO Schendel SL Fusco ML Gangavarapu K Gunn BM Wec AZ. Systematic Analysis of Monoclonal Antibodies Against Ebola Virus GP Defines Features That Contribute to Protection. Cell (2018) 174(4):938–952 e13. 10.1016/j.cell.2018.07.033 30096313 PMC6102396

[27] Gunn BM Lu R Slein MD Ilinykh PA Huang K Atyeo C. A Fc Engineering Approach to Define Functional Humoral Correlates of Immunity Against Ebola Virus. Immunity (2021) 54(4):815–28.e5. 10.1016/j.immuni.2021.03.009 33852832 PMC8111768

[28] Karsten CB Mehta N Shin SA Diefenbach TJ Slein MD Karpinski W. A Versatile High-Throughput Assay to Characterize Antibody-Mediated Neutrophil Phagocytosis. J Immunol Methods (2019) 471:46–56. 10.1016/j.jim.2019.05.006 31132351 PMC6620195

[29] Ackerman ME Moldt B Wyatt RT Dugast AS McAndrew E Tsoukas S. A Robust, High-Throughput Assay to Determine the Phagocytic Activity of Clinical Antibody Samples. J Immunol Methods (2011) 366(1-2):8–19. 10.1016/j.jim.2010.12.016 21192942 PMC3050993

[30] Fischinger S Fallon JK Michell AR Broge T Suscovich TJ Streeck H. A High-Throughput, Bead-Based, Antigen-Specific Assay to Assess the Ability of Antibodies to Induce Complement Activation. J Immunol Methods (2019) 473:112630. 10.1016/j.jim.2019.07.002 31301278 PMC6722412

[31] Brown EP Licht AF Dugast AS Choi I Bailey-Kellogg C Alter G. High-Throughput, Multiplexed IgG Subclassing of Antigen-Specific Antibodies From Clinical Samples. J Immunol Methods (2012) 386(1-2):117–23. 10.1016/j.jim.2012.09.007 PMC3475184 23023091

[32] Lin A Salvador A Carter JM. Multiplexed Microsphere Suspension Array-Based Immunoassays. Methods Mol Biol (2015) 1318:107–18. 10.1007/978-1-4939-2742-5_11 26160569

[33] Halfmann PJ Eisfeld AJ Watanabe T Maemura T Yamashita M Fukuyama S. Serological Analysis of Ebola Virus Survivors and Close Contacts in Sierra Leone: A Cross-Sectional Study. PloS Negl Trop Dis (2019) 13(8):e0007654. 10.1371/journal.pntd.0007654 31369554 PMC6692041

[34] Thom R Tipton T Strecker T Hall Y Akoi Bore J Maes P. Longitudinal Antibody and T Cell Responses in Ebola Virus Disease Survivors and Contacts: An Observational Cohort Study. Lancet Infect Dis (2020) 21(4):507–16. 10.1016/S1473-3099(20)30736-2 PMC7553754 33065039

[35] Mulangu S Alfonso VH Hoff NA Doshi RH Mulembakani P Kisalu NK. Serologic Evidence of Ebolavirus Infection in a Population With No History of Outbreaks in the Democratic Republic of the Congo. J Infect Dis (2018) 217(4):529–37. 10.1093/infdis/jix619 PMC5853806 29329455

[36] Smiley Evans T Tutaryebwa L Gilardi KV Barry PA Marzi A Eberhardt M. Suspected Exposure to Filoviruses Among People Contacting Wildlife in Southwestern Uganda. J Infect Dis (2018) 218(suppl_5):S277–86. 10.1093/infdis/jiy251 PMC6927880 29924324

[37] Becquart P Wauquier N Mahlakoiv T Nkoghe D Padilla C Souris M. High Prevalence of Both Humoral and Cellular Immunity to Zaire Ebolavirus Among Rural Populations in Gabon. PloS One (2010) 5(2):e9126. 10.1371/journal.pone.0009126 20161740 PMC2817732

[38] Brenchley JM Price DA Schacker TW Asher TE Silvestri G Rao S. Microbial Translocation is a Cause of Systemic Immune Activation in Chronic HIV Infection. Nat Med (2006) 12(12):1365–71. 10.1038/nm1511 17115046

[39] McElroy AK Akondy RS Davis CW Ellebedy AH Mehta AK Kraft CS. Human Ebola Virus Infection Results in Substantial Immune Activation. Proc Natl Acad Sci USA (2015) 112(15):4719–24. 10.1073/pnas.1502619112 PMC4403189 25775592

[40] Adaken C Scott JT Sharma R Gopal R Dicks S Niazi S. Ebola Virus Antibody Decay-Stimulation in a High Proportion of Survivors. Nature (2021) 590(7846):468–72. 10.1038/s41586-020-03146-y PMC7839293 33505020

[41] Sudre CH Murray B Varsavsky T Graham MS Penfold RS Bowyer RC. Attributes and Predictors of Long COVID. Nat Med (2021) 27(4):626–31. 10.1038/s41591-021-01292-y PMC7611399 33692530

[42] Nalbandian A Sehgal K Gupta A Madhavan MV McGroder C Stevens JS. Post-Acute COVID-19 Syndrome. Nat Med (2021) 27(4):601–15. 10.1038/s41591-021-01283-z PMC8893149 33753937

[43] Ahmed H Patel K Greenwood DC Halpin S Lewthwaite P Salawu A. Long-Term Clinical Outcomes in Survivors of Severe Acute Respiratory Syndrome and Middle East Respiratory Syndrome Coronavirus Outbreaks After Hospitalisation or ICU Admission: A Systematic Review and Meta-Analysis. J Rehabil Med (2020) 52(5):jrm00063. 10.2340/16501977-2694 32449782

[44] Tritsch SR Encinales L Pacheco N Cadena A Cure C McMahon E. Chronic Joint Pain 3 Years After Chikungunya Virus Infection Largely Characterized by Relapsing-remitting Symptoms. J Rheumatol (2020) 47(8):1267–74. 10.3899/jrheum.190162 PMC7938419 31263071

