# Supplementary material for: Associations Between Antibody Fc-Mediated Effector Functions and Long-Term Sequelae in Ebola Virus Survivors
Source: Front Immunol. 2021 May 20;12:682120. doi: 10.3389/fimmu.2021.682120 (PMC8173169; doi:10.3389/fimmu.2021.682120)
Supplement: Supplementary file 1 [file Table_1.docx]

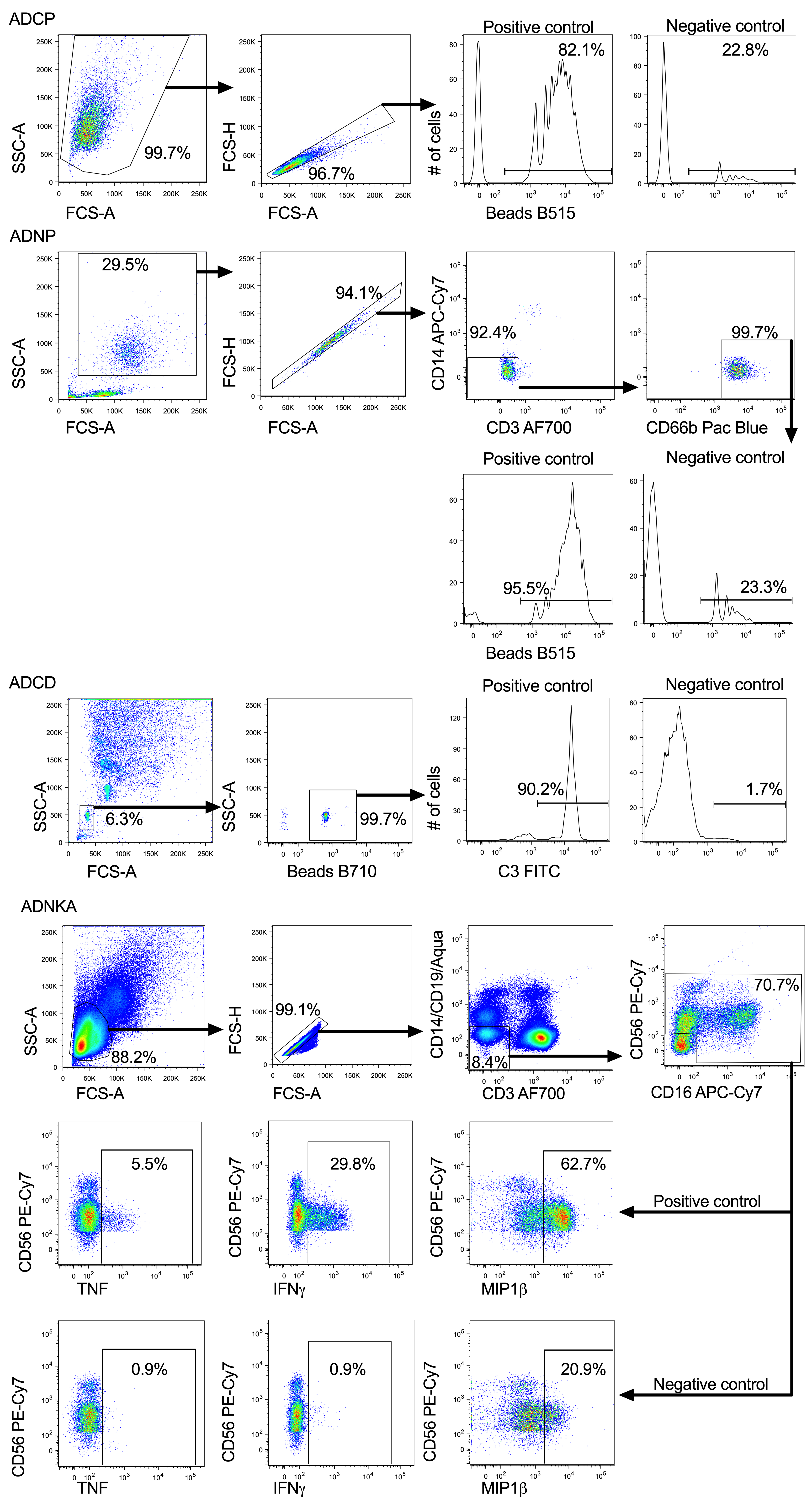


Supplementary Figure 1. Gating stategy and representative flow plots for ADCP, ADNP, ADCD, and ADNKA.


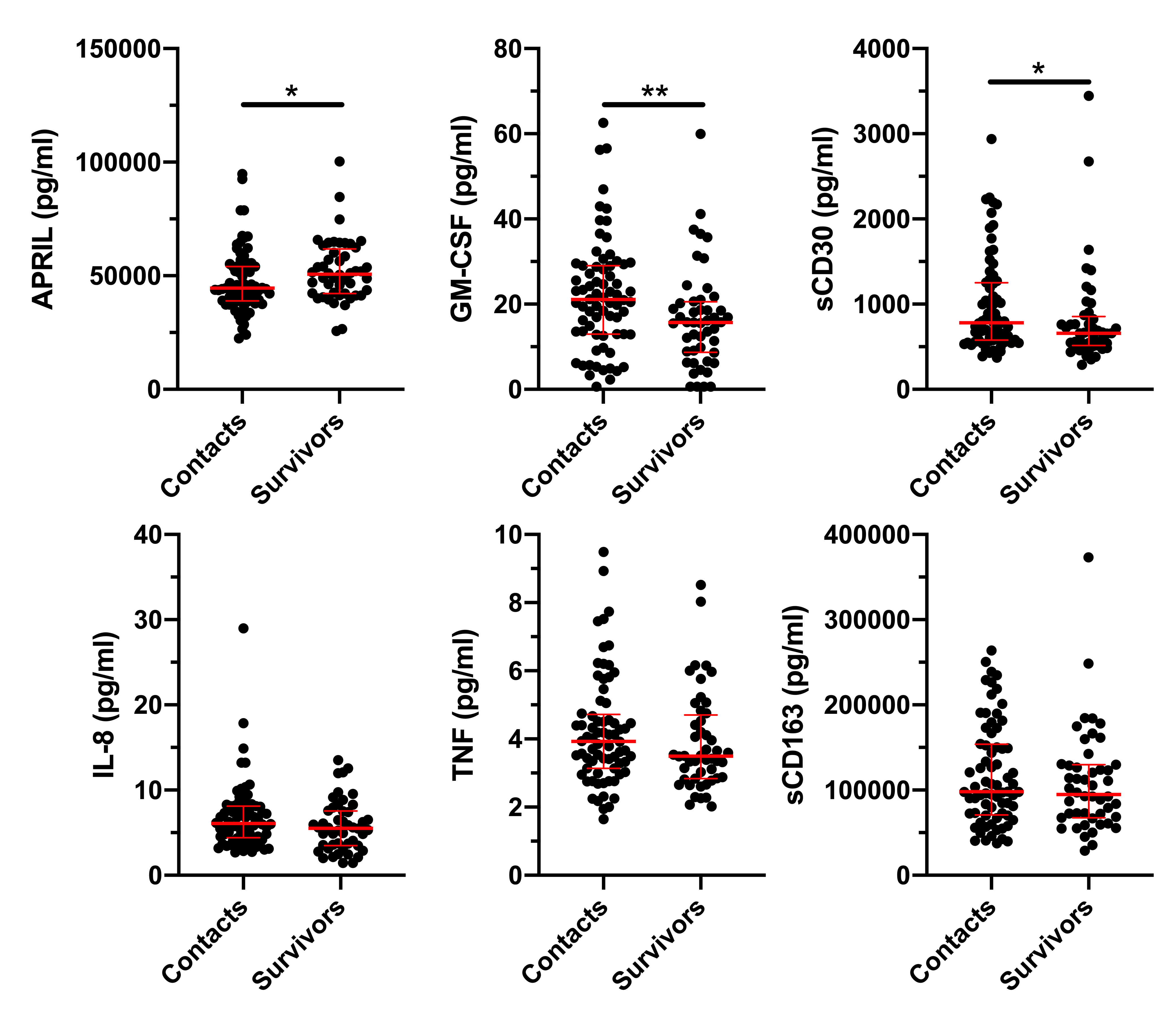


Supplementary Figure 2. Plasma levels of APRIL, GM-CSF, sCD30, IL-8, TNF, and sCD163 in BDBV survivors (n=48) and household contacts (n=72). The line and whiskers represent the median and interquartile range respectively. ** represents p values < 0.01 and * represents p values < 0.05.


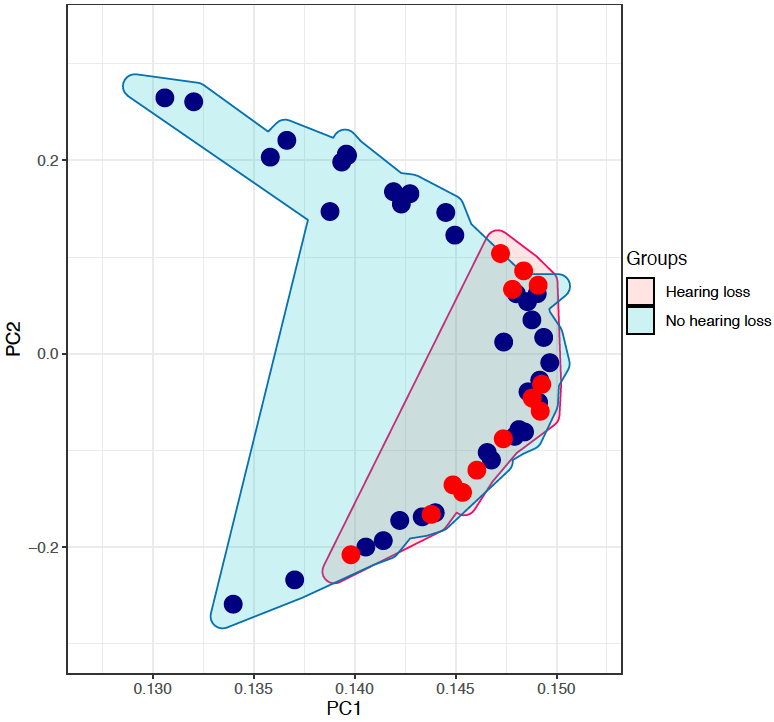


Supplementary Figure 3. Principal Component Analysis (PCA) of the soluble markers measured in BDBV survivors, with individuals with (red) or without (blue) hearing loss plotted against Principal Component (PC) 1 and PC2.
